# Can we learn from errors? Retrieval facilitates the correction of false memories for pragmatic inferences

**DOI:** 10.1371/journal.pone.0272427

**Published:** 2022-08-02

**Authors:** María J. Maraver, Ana Lapa, Leonel Garcia-Marques, Paula Carneiro, Ana Raposo

**Affiliations:** Faculdade de Psicologia, CICPSI, Universidade de Lisboa, Lisbon, Portugal; University of Zurich, SWITZERLAND

## Abstract

Errorful learning suggests that, when perfect learning has not yet been attained, errors can enhance future learning if followed by corrective feedback. Research on memory updating has shown that after retrieval, memory becomes more malleable and prone to change. Thus, retrieval of a wrong answer might provide a good context for the incorporation of feedback. Here, we tested this hypothesis using sentences including pragmatic sentence implications, commonly used for the study of false memories. Across two experiments with young adults, we hypothesized that corrective feedback would be more efficient at reducing false memories if provided immediately after retrieval, when memory is more malleable than after being exposed to the material. Participants’ memory was assessed as a function of the type of learning task (Experiment 1: retrieval vs. restudy; and Experiment 2: active vs. passive recognition); and whether participants received corrective feedback or not. In both experiments, we observed that retrieval not only improved correct recall (replicating the testing effect) but also promoted the correction of false memories. Notably, corrective feedback was more effective when given after errors that were committed during retrieval rather than after restudy (Experiment 1) or after passive recognition (Experiment 2). Our results suggest that the benefits of retrieval go beyond the testing effect since it also facilitates false memories correction. Retrieval seems to enhance memory malleability, thus improving the incorporation of feedback, compared to the mere presentation of the information. Our results support the use of learning strategies that engage in active and explicit retrieval because, even if the retrieved information is wrong—when immediate feedback is provided—memory updating is promoted and errors are more likely to be corrected.

## Introduction

Making errors is uncomfortable, even though we frequently make them. Especially in educational settings, educators may be reluctant to ask students to guess before learning the correct information for fear that their incorrect guesses would be confused with true items and be harmful to future learning. On the other hand, many theories of associative and predictive learning [1,2] assume that learning can only occur after performance or predictive errors (followed by accurate feedback). What is the most optimal strategy for promoting proper learning is still an unresolved question: whether and in what conditions, if any, it is better to avoid making errors than to commit, explore, and correct mistakes [3]. This study aimed to explore the consequences of making errors during the learning process and to contribute to the current debate on whether and under which conditions, generating errors can be beneficial or detrimental to learning.

Retrieving previously learned information from memory has been widely evidenced as one of the most effective learning strategies. This robust finding, known as the “testing effect” or “retrieval practice effect”, shows that when learned information is retrieved by taking memory tests, it is more likely to be correctly recalled in the future than when the information is reread or restudied [4,5]. The testing effect has been highly replicated both in the laboratory and school settings. Yet, these studies have traditionally focused on successful retrieval [4–7], that is, when the retrieved information is correct. Much less is known about the impact of unsuccessful retrieval on subsequent learning. When a wrong answer is given in a memory test, it remains unclear what happens to the subsequent retrievability of the correct answer.

In the last two decades, an increasing number of studies have shown that generating errors, as long as followed by feedback, can enhance later memory for the correct response [3,8,9]. These results contradict classic interference theories that suggest that a generated wrong response might interfere and hinder the learning of the correct answer because of an enhanced memorability of the erroneous response [10,11]. This rationale is supported by traditional theories of learning and memory that advocate that errors should be avoided, and teaching should follow an *errorless learning* procedure encouraging students to make as few errors as possible [10,12–14].

However, associative and predictive learning models argue that the revision of associative weights (associative learning) between predictive and criterium items are a function of performance or predictive errors, such that learning cannot improve without errors [1,2]. Early studies by Izawa [15,16] introduced the hypothesis that in comparison to errorless learning, the generation of errors, as long as it is followed by corrective feedback, results in better memory for the correct response. These *errorful learning* perspectives propose that failed retrieval attempts can improve learning given that the occurring mismatch between the wrong response and the corrective feedback would increase attention to the encoding of the feedback and, therefore, strengthen learning [9]. When an error is committed, providing feedback with the availability of the correct answer is paramount to process, understand and integrate the corrected information into memory [17,18].

Various accounts have been put forward to explain the benefits of error generation and error correction. Retrieving a memory trace, even if wrong, could potentially strengthen retrieval pathways to related content, identifying a need for additional information, and turning memory into a fertile ground susceptible to the encoding of new information [19]. According to the reconsolidation hypothesis, retrieving a consolidated memory can increase its malleability within a limited time window, in which the memories become susceptible to modification [20,21]. One of the most adaptive properties of our memory is its function to update and revise the contents. In this sense, memory updating allows our memory to stay current and up to date, eliminating information that is no longer needed to avoid error perpetuation [22]. The Memory Updating After Retrieval (MUAR) framework explains how retrieval is a strong modifier of memory. It proposes that retrieval attempts make memory more malleable, promoting an optimal environment for the updating of information relevant for retrieval, in this case, the incorporation of the feedback [22]. The finding that knowledge can be successfully updated after self-generated errors has been demonstrated with experimental paradigms such as last word generation in sentences [23], word-definition or translation pairs [9,24], generation of antonyms [25], or related word pairs [26–29], on immediate and delayed follow-up tests. Kornell and colleagues demonstrated that producing errors led to better learning in comparison to merely being exposed to the correct response using the standard error-generation paradigm with different materials for cue-target pairs (i.e., general-knowledge questions or weak associates word pairs). Across experiments, the authors found that guessing a wrong answer before being provided with corrective feedback enhanced learning on a final memory test, compared to conditions in which the question and the answer were simultaneously presented [26]. Such a beneficial effect of error generation has been replicated with similar guessing errors that were semantically related to the correct answer [29–33]. The guessing methodology has the advantage of ensuring a high number of unsuccessful retrieval attempts. However, guesses do not represent all types of errors, and in most cases, responses are only right or wrong in the context of the experiment. For example, in the weak-associate paradigm, the generation of the strong associate after the presentation of the cue (e.g., snow-white) is often considered a guessing error, whereas the cue-weak associate pair (e.g., snow-balls) represents the correct response. Therefore, these types of mistakes are not genuine generated errors, but arbitrary guesses. The differences between guessing and retrieval may be of great importance to the understanding of the beneficial effects of error correction. According to the semantic mediation hypothesis, the benefit of error generation occurs when the error can act as a semantic mediator, thereby increasing the retrievability of the correct item and facilitating the target retrieval (see for instance, [34,35]). This explanation would account for the finding that, when the guessing error and the target are semantically related, retrieval is enhanced [26,32,35–37]. Other research however has found no effect or even a detrimental effect of guessing when unrelated word pairs were learned [30,31,38–40]. As Metcalfe and Huesler [28] argue: “the notion that the benefit seen from the generation of errors in typical participants is attributable to semantic memory mediation sits awkwardly with the findings that amnesics who are thought to have intact semantic memory but not episodic memory [41–43] do not similarly benefit” [28]. Further limitations of the semantic mediation hypothesis come from Metcalfe and Huesler [28] who showed that guessing errors that were semantically incongruent with the target (i.e., “tree-palm” for the target “hand” vs. “wrist-palm” as the congruent condition), still provided performance benefits as long as both (error and item) were recalled. Thus, an explanation in terms of semantic mediation is clearly incomplete and a more comprehensive interpretation must include the role of episodic recollection. That is, when information is retrieved from memory (even if it is wrong), the answer is computed through the search and navigation into a richer episodic network, probably requiring a more elaborated form of processing than the one underlying (uninformed) guessing.

Because of the limitations of guessing errors, it remains unexplored what the benefits of error correction are when using materials that generate responses that involve an episodic retrieval of a previous context or a learning event. This question is key to understand the benefits of error correction given that research has suggested that a guessing error is only beneficial to learning when it can be used as a semantic mediator [26,32,36,37] but not when unrelated word pairs are learned [30,31,38,39]. However, the MUAR framework suggests that when information is retrieved from memory (even if incorrectly), memory becomes more vulnerable and amenable to change, making it more prone to be updated with new information [22] –as would be the case of corrective feedback–suggesting that retrieval might be a promotor for error correction. Carneiro and colleagues provided evidence for this hypothesis, using DRM lists that required the study of semantically related words (e.g., “bed, tired, rest, pillow, dream”), that often led to falsely remembering a non-studied critical lure (e.g., “sleep”) at a later memory test. The authors compared the effect of presenting new information, (i.e., corrective feedback) when provided after retrieval vs. after restudy [44]. Consistent with the MUAR framework, their results suggested that retrieval followed by feedback is a better learning environment than restudy. But even if this result was found using a paradigm that involved episodic retrieval, it disregarded two important aspects. First, retrieval and restudy practices were not assessed when no corrective feedback was given, making it difficult to disentangle what is the actual impact that providing corrective feedback had on learning practices. And second, previous research has shown that the associative memory errors generated with the DRM lists correlate poorly with other types of memory errors such as false-event suggestion (remembering an event that did not happen) or misinformation (updating a previous memory with false information), that involve the episodic recall of real-life events [45]. Therefore, it becomes critical to extend the results obtained by Carneiro et al. [44] and study the effect of retrieval on error correction but using materials that involve episodic retrieval and are more ecologically valid.

The present work attempted to overcome these limitations and to address in-depth the effect of retrieval followed by feedback on the correction of memory errors, by adopting a paradigm with greater ecological validity. Sentences including pragmatic implications are a useful tool as they involve an episodic retrieval of a previous context or a learning event and represent more closely the type of errors that occur in real-world situations. For example, the sentence “*The boy lost balance on the skate*” is usually falsely remembered as “*The boy fell from the skate*”, or “*The baby stayed awake all night*” is very often misremembered as “*The baby cried all night*”, when in both cases the remembered outcomes were never explicitly presented [46]. Pragmatic inferences have been widely used for the study of false memories for everyday actions since memory errors are easily created, vivid, and held with high confidence [46–48]. Importantly, they allow for the dissociation between a semantic (i.e., the concepts of baby and to cry are semantically related), and an episodic component (i.e., the explicitly presented sentence “*The baby stayed awake all night*”). Therefore, by using pragmatic inferences we could evaluate the generation of errors that stem from episodic retrieval, and the effects of their correction on subsequent learning.

We tested the hypothesis of whether the retrieval of memory errors generated from pragmatic inferences could improve learning compared to conditions in which the information was merely presented, while contrasting the presence and absence of corrective feedback. Two experimental studies with young adults were conducted using pragmatic inference sentences in Portuguese [47]. Both experiments included a first learning encoding phase, a second intermediate phase in which our key manipulations were applied, and a final cued-recall memory test in which learning was assessed. In Experiment 1, we compared the effects of the learning task (retrieval vs. restudy) and the presentation of feedback (present vs. absent). During the intermediate phase of Experiment 1, participants either performed a cued-recall retrieval task in which the fragment that triggered the pragmatic inference of the sentence had to be completed (i.e., *“The baby _____ all night*.*”*); or a restudy task in which half of the sentences were presented in their correct form (i.e., the same sentence that was presented during encoding *“The baby stayed awake all night”*) and the other half in an incorrect form (i.e., different from encoding *“The baby cried all night”*). As for the feedback manipulation, Mullet & Marsh suggest that two factors are essential to correct false memories [49]. First, the learner needs to realize a mistake has been made. Therefore, feedback should be presented immediately after an error is committed to allow learners to notice the discrepancy between the error and the correct information. And second, there is empirical and theoretical consensus that for feedback to be effective, learners need to know the correct information and not only that a mistake was made [18,22], so that explicit corrective feedback is essential to promote successful error correction [49]. Thus, in the current experiments, when corrective feedback was provided, it was presented immediately after the responded or restudied sentence, and it contained the correct information, (i.e., the sentence studied at the initial encoding phase).

Retrieval and restudy conditions of Experiment 1 led to an unbalanced proportion errors and to different types of errors across conditions. Specifically, in the retrieval condition, the proportion of errors generated varied across participants, whereas in the restudy condition, for all participants, 50% of the sentences presented contained an error. Therefore, to increase experimental control and to even the errors’ ratio that participants committed versus were exposed to, we conducted a second experiment in which we implemented a yoked design and used a recognition instead of a cued-recall task in the intermediate phase. In Experiment 2, experimental conditions were compared as a function of feedback (present vs. absent as in Experiment 1), and the recognition paradigm (active vs. passive). Participants in the active recognition condition were presented with sentences that could match or mismatch the sentences presented at encoding and had to respond whether it was correct (same as encoding) or incorrect (different from encoding), as in a typical recognition test. Participants in the passive recognition condition were simply instructed to read the stimuli that was about to be presented. They were presented with the same sentences that participants in the active condition saw, but instead of having to provide an answer, they were shown the responses that a participant in the active condition had given. Lastly, in both Experiments 1 and 2, participants completed a final cued-recall test where the proportion of correct responses, pragmatic inferences errors, intrusions and omissions were assessed to measure the impact that the learning conditions and feedback had on memory performance.

For both studies, we hypothesized that, overall, retrieval would lead to a better final memory performance for sentences correctly recalled at the intermediate phase thus, replicating the testing effect [5]. Moreover, we anticipated that, when no feedback was provided, retrieval would lead to an enhancement of false memory errors compared to the conditions in which the information was merely presented (restudy for Experiment 1, and passive recognition for Experiment 2). However, when corrective feedback was provided, we predicted retrieval at the intermediate phase to decrease false memory errors in the final memory test. Because after retrieval memory seems to be more prone to change [22], even when the retrieved information is incorrect, it might result in a deeper and more elaborated processing which, in turn, would lead to a richer context for encoding the corrective feedback. Henceforth, we expected corrective feedback to be better incorporated into the retrieved memories, than when it is presented after information that was restudied (Experiment 1) or passively recognized (Experiment 2).

## Experiment 1

### Methods

#### Participants

The sample size was determined according to sample resources availability on a priori grounds—based on previous studies with similar experimental protocols manipulating error correction [44] or using pragmatic inferences as experimental material [50]. 120 university students from the University of Lisbon participated in the experiment (*M*_age_ = 21.05 ± 6.24; 100 female) and were rewarded with 10€ vouchers for their participation. Participants were randomly assigned to one out of the four experimental conditions by crossing the two manipulated factors: learning condition (retrieval (*n* = 30) vs. restudy (*n* = 30)), and corrective feedback (present (*n* = 30) vs. absent (*n* = 30)).

All experimental procedures were approved by the Ethics Committee of the Faculty of Psychology of the University of Lisbon. Prior to the start of the experimental session, participants received information about the study and provided written consent for their participation in accordance with the Declaration of Helsinki [51].

#### Materials

Sixty sentences including pragmatic inferences in Portuguese from Carneiro et al. [47] were previously pretested to assess the proportion of pragmatic inferences falsely recalled. The thirty sentences with the highest proportion of false recall were selected and used in the current study. These thirty sentences were pretested again to assess their average proportion of pragmatic inferences when only thirty sentences composed the task. Pragmatic inferences for these sentences were generated, on average, 54% of the time.

#### Design

This experiment followed a 2 (learning condition: retrieval vs. restudy) × 2 (corrective feedback: present vs. absent) factorial design, with both variables manipulated between participants.

#### Procedure

Participants performed the task individually on computers at the laboratory of the Faculty of Psychology of the University of Lisbon. The experimental task included three separate phases, and the procedure is illustrated in Table 1. During the encoding phase, all participants were instructed to read the sentences presented on the computer screen and to memorize them (e.g., *The baby stayed awake all night*). Sentences were presented in a black font, for 4.5 seconds, in random order. After the presentation of each sentence, participants had 5 seconds to perform an easy arithmetic operation (e.g., 23–5 = ?) to prevent retrieval, as used in previous protocols [50]. Before the start of the task, participants completed a practice trial with five sentences (without pragmatic implications). After the encoding phase, participants performed a distractor task for 5 minutes, where they were asked to count the differences between 4 pairs of images.

**Table 1:** Procedure scheme of Experiment 1.

**Encoding phase**				
*The baby stayed awake all night* 5 + 4 = 12				
**Intermediate phase**				
Task	Retrieval	*The baby ____ all night*	Feedback	*The baby stayed awake all night*
			No feedback	23–7 =
	Restudy	*The baby cried all night*	Feedback	*The baby stayed awake all night*
			No feedback	36 + 6 =
**Final cued-recall test**				
*The baby ____ all night*				

Afterwards, participants completed the intermediate phase in which the key manipulations were applied. In the retrieval condition, participants performed a cued-recall test: each sentence from encoding was now presented in a random order with a critical fragment missing (e.g., *The baby ____ all night*). This cue was presented for 10.5 seconds in blue font. Participants were asked to complete the missing fragment with the information presented at encoding and had a maximum of 20 seconds to provide an answer. In the restudy condition, participants were told that they would be presented with the recall output of another person from a previous experimental session, and their task was simply to read that sentence. Participants were presented with sentences from encoding, in a random order, but to mimic the errors that participants in the retrieval condition are expected to make at this phase, 50% of the presented sentences were correct (presented in the same format as encoding) whereas the other 50% were incorrect (presented in a different format from encoding). This ratio of correct to incorrect sentences matched approximately the percentage of pragmatic inference errors expected for those in the retrieval condition, based on the pilot study for the same sentences (0.54 rate of false recall). The correct/incorrect format of each sentence was counterbalanced across participants. Each sentence was presented for 10.5 seconds in blue font. Across both conditions, after retrieval or restudy of each sentence, participants in the feedback condition, were provided with corrective feedback: the correct sentence (i.e., in the same format as encoding) was presented for 4.5 seconds, in a black font, matching the color of encoding; participants in the no feedback condition were not presented with the correct sentence, instead, performed an easy arithmetic operation after each sentence for 4.5 seconds. After the intermediate phase, all participants performed another distractor task, like the previous one, for 1 minute. Finally, all participants performed a final cued-recall test in which all 30 sentences from encoding were presented with the critical fragment missing, and to be completed (i.e., *The baby ____ all night*). The sentences were presented in a random order, participants had 60 seconds to provide each answer and no feedback was provided. After completing the final cued-recall test, participants were thanked and rewarded for their time.

#### Statistical analyses

The list of experimental sentences used and the corresponding coding criteria for correct responses and pragmatic inference errors can be found in the supplementary material. Like in previous protocols [50], responses at the intermediate cued-recall test for those in the retrieval condition, and in the final cued-recall test for all participants were recoded following an adaptation of the standard coding procedure from Brewer [46], resulting in four response types: i) correct responses, which corresponded to those answers matching the original sentence or synonyms maintaining the original meaning of the sentence (i.e., “*put his lips towards”*, *“approached*” for the sentence “*The charming prince gently put his lips towards Snow White’s cheek*”); ii) pragmatic inference responses, which included responses that matched the expected pragmatic inferences or their synonyms (i.e., “*kissed*”), meeting the but-not test (i.e., “*The charming prince gently put his lips towards Snow White’s cheek*, *but did not kiss her*”); iii) intrusions, corresponding to other alternative answers (i.e., “*touched”*, *“smelled”*,*”looked*”); and iv) omissions, for responses left blank. The proportion of each response type was calculated, resulting in four dependent variables.

To compare memory retrieval at the intermediate phase between participants in the feedback and no feedback conditions, independent samples *t*-tests were performed over the proportion of recall of the four response types (correct responses, pragmatic inferences, intrusions, and omissions). Next, between-subjects ANOVAs were performed over the proportion of recall of each response type as a function of the learning condition (retrieval vs. restudy) and the presence of feedback at the intermediate phase (present vs. absent). Finally, two indexes were calculated as a function of the responses given at the intermediate and final cued-recall phases in order to analyze the persistence of correct responses and the correction of errors. Analyses were run using IBM SPSS Statistics software version 25 (IBM Corp., Armonk, NY, United States).

### Results

#### Memory performance at the intermediate phase

At the intermediate phase, we observed no differences in memory performance between participants that did and did not receive feedback after the retrieval task (see Table 2).

**Table 2:** Memory performance at the retrieval condition of the intermediate phase.

	Feedback	No feedback	*t*(58)	*p*	95% Confidence Interval
**Correct responses**	0.29 ±0.15	0.23±0.11	1.74	0.87	[-0.01–0.13]
**Pragmatic inferences**	0.61±0.15	0.64±0.11	0.95	0.34	[-0.10–0.03]
**Intrusions**	0.04±0.07	0.08±0.07	1.87	0.07	[-0.07–0.00]
**Omissions**	0.06±0.05	0.05±0.05	0.68	0.50	[-0.02–0.03]

Descriptive statistics (mean ± standard deviations) and independent samples *t*-test statistics for each response type in the feedback and no feedback groups of the retrieval condition at the intermediate phase.

#### Memory performance at the final cued-recall test

Table 3 summarizes the proportion of cued-recall at the final test for each experimental condition and response type for Experiments 1 and 2.

**Table 3:** Memory performance at the final cued-recall test.

**Correct responses**						
	Experiment 1			Experiment 2		
	Retrieval	Restudy	Total	Active recognition	Passive recognition	Total
Feedback	0.83±0.10	0.61±0.21	0.72±0.20	0.71±0.19	0.51±0.28	0.61±0.26
No feedback	0.22±0.12	0.32±0.12	0.28±0.13	0.27±0.15	0.23±0.17	0.25±0.16
Total	0.53±0.33	0.47±0.22	0.50±0.28	0.50±0.30	0.37±0.27	0.43±0.28
**Pragmatic inferences**						
	Experiment 1			Experiment 2		
	Retrieval	Restudy	Total	Active recognition	Passive recognition	Total
Feedback	0.14±0.09	0.33±0.19	0.23±0.17	0.22±0.16	0.40±0.25	0.31±0.22
No feedback	0.67±0.14	0.63±0.14	0.65±0.14	0.64±0.13	0.65±0.17	0.65±0.15
Total	0.40±0.29	0.48±0.22	0.44±0.26	0.43±0.25	0.52±0.25	0.47±0.25
**Intrusions**						
	Experiment 1			Experiment 2		
	Retrieval	Restudy	Total	Active recognition	Passive recognition	Total
Feedback	0.01±0.02	0.03±0.03	0.02±0.03	0.05±0.05	0.08±0.07	0.07±0.06
No feedback	0.06±0.07	0.03±0.04	0.05±0.06	0.08±0.08	0.08±0.07	0.08±0.08
Total	0.04±0.05	0.03±0.03	0.04±0.05	0.06±0.07	0.08±0.07	0.07±0.07
**Omissions**						
	Experiment 1			Experiment 2		
	Retrieval	Restudy	Total	Active recognition	Passive recognition	Total
Feedback	0.02±0.03	0.03±0.08	0.02±0.06	0.01±0.03	0.01±0.03	0.01±0.03
No feedback	0.04±0.07	0.02±0.04	0.03±0.05	0.00±0.02	0.03±0.08	0.02±0.06
Total	0.03±0.05	0.02±0.06	0.03±0.06	0.01±0.03	0.02±0.06	0.01±0.05

Descriptive statistics (mean ± standard deviations) of the four response types assessed at the final cued-recall test as a function of the experimental conditions: Feedback vs. no feedback and learning condition (retrieval vs. restudy for Experiment 1, and active vs. passive recognition for Experiment 2).

Results of the ANOVA revealed a higher proportion of correct responses in the final cued-recall test for the retrieval condition compared to the restudy (*F*(1,116) = 4.97, *p* = 0.03, *η*^*2*^_*p*_ = 0.04), replicating the testing effect. The presentation of feedback also resulted in more correct responses compared to not receiving feedback (*F*(1,116) = 282.88, *p* < 0.01, *η*^*2*^_*p*_ = 0.71). And a significant interaction between the retrieval task and feedback (*F*(1,116) = 36.28, *p* < 0.01, *η*^*2*^_*p*_ = 0.24) showed that the beneficial effect of giving feedback was larger for retrieval (*F*(1,58) = 440.82, *p <* 0.01, *η*^*2*^_*p*_ = 0.88) than for the restudy condition (*F*(1,58) = 41.38, *p <* 0.01, *η*^*2*^_*p*_ = 0.42).

A similar pattern of results was observed for pragmatic inference errors. Participants who performed the retrieval task during the intermediate phase generated a lower proportion of pragmatic inference errors at the final test than those who restudied the material (*F*(1,116) = 7.92, *p* < 0.01, *η*^*2*^_*p*_ = 0.06). Receiving feedback similarly reduced the proportion of pragmatic inferences compared to no feedback (*F*(1,116) = 254.50, *p* < 0.01, *η*^*2*^_*p*_ = 0.69). The significant interaction (*F*(1,116) = 20.44, *p* < 0.01, *η*^*2*^_*p*_ = 0.15) revealed that the reduction of pragmatic inference errors following feedback was greater for the retrieval (*F*(1,58) = 322.63, *p* < 0.01, *η*^*2*^_*p*_ = 0.85) than for the restudy condition (*F*(1,58) = 48.39, *p* < 0.01, *η*^*2*^_*p*_ = 0.45).

For intrusions, we did not observe a main effect of retrieval task (*F*(1,116) = 1.26, *p* = 0.26, *η*^*2*^_*p*_ = 0.01), but there was a main effect of feedback (*F*(1,116) = 11.33, *p* < 0.01, *η*^*2*^_*p*_ = 0.09) and an interaction (*F*(1,116) = 7.10, *p* < 0.01, *η*^*2*^_*p*_ = 0.06). This indicates that while for the restudy group there was no difference in the proportion of intrusions in the feedback and no feedback conditions (*F*(1,58) = 0.37, *p =* 0.55, *η*^*2*^_*p*_ < 0.01), for the retrieval group there were fewer intrusions in the feedback compared to the no feedback condition (*F*(1,58) = 13.66, *p* < 0.01, *η*^*2*^_*p*_ = 0.19).

Lastly, for omissions, we did not observe a main effect of retrieval task (*F*(1,116) = 0.0.22, *p =* 0.64, *η*^*2*^_*p*_ < 0.01) or feedback (*F*(1,116) = 0.32, *p =* 0.57, *η*^*2*^_*p*_ < 0.01), but there was an interaction between the factors (*F*(1,116) = 4.08, *p =* 0.05, *η*^*2*^_*p*_ = 0.03). While no difference was observed between feedback and no feedback for the restudy conditions (*F*(1,58) = 0.88, *p =* 0.35, *η*^*2*^_*p*_ = 0.01), presenting feedback after retrieval led to fewer omissions than having no feedback (*F*(1,58) = 4.14, *p =* 0.05, *η*^*2*^_*p*_ = 0.07).

#### Persistence of correct responses and error correction

**Fig 1:**
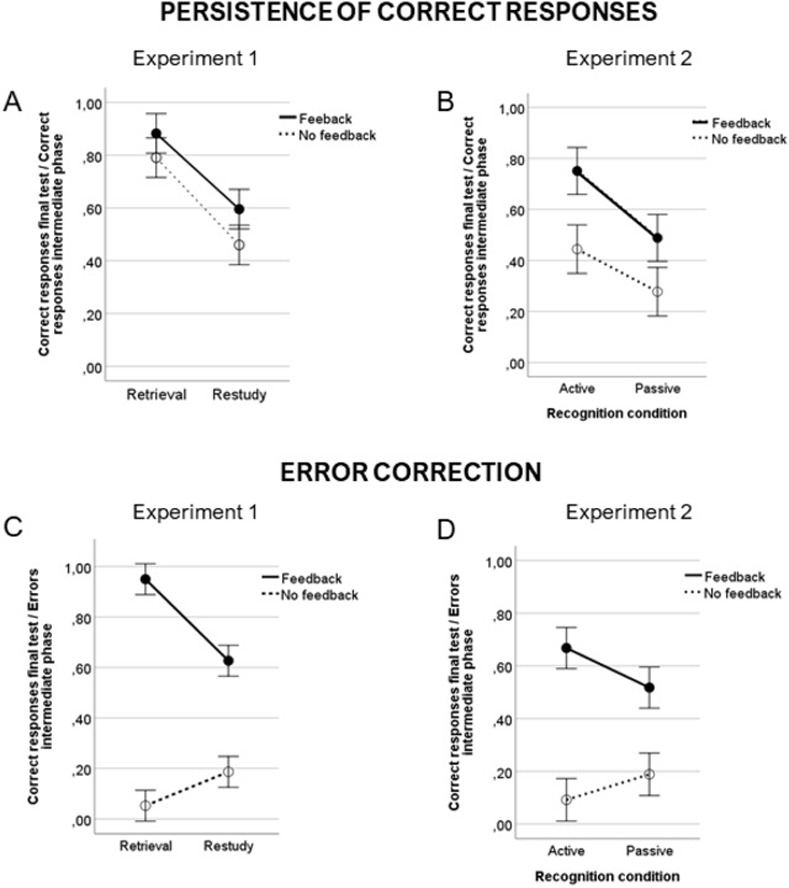
Memory performance. Memory indexes calculated from performance at the final cued-recall test as a function of performance in the intermediate phase. A, B: Persistence of correct responses; C, D: Error correction. Left panels depict data for Experiment 1 and right panels represent performance in Experiment 2.

To further investigate the effects of the experimental manipulations and to directly test our hypotheses, we analyzed memory performance at the final cued-recall test as a function of participants responses during the intermediate phase. To that purpose, we calculated two different memory indexes (see Fig 1) and introduced them as dependent variables in a 2×2 between subjects ANOVA with learning condition (retrieval vs. restudy) and feedback (present vs. absent) as factors.

To assess the persistence of correct responses, we considered the sentences that had been recalled/restudied correctly at the intermediate phase and inspected if they continued to be correctly recalled in the final test. We calculated this index through the sum of correct responses in the final test that had been correctly recalled/restudied in the intermediate phase divided by the total number of sentences correctly recalled/presented in the intermediate phase. Results showed that participants that performed the retrieval task had a higher rate of persistence of correct responses (*M* = 0.83 ± 0.22) compared to those who restudied the sentences (*M* = 0.53 ± 0.21; *F*(1,116) = 66.47, *p <* 0.01, *η*^*2*^_*p*_ = 0.07), replicating the testing effect. Receiving feedback also led to a greater persistency of the correct responses (*M* = 0.74 ± 0.25) than not receiving feedback (*M* = 0.62 ± 0.27, *F*(1,116) = 9.00, *p <* 0.01, *η*^*2*^_*p*_ = 0.07). We did not observe an interaction between the factors (*F*(1,116) = 0.33, *p =* 0.57, *η*^*2*^_*p*_ < 0.01, see Fig 1A).

The most interesting measure of our analysis was the assessment of error correction in those sentences that, at the intermediate phase, were incorrectly recalled/restudied but at the final cued-recall test were correctly recalled. We computed this index as the sum of correct responses in the final test phase that had previously been pragmatic inferences errors in the intermediate phase divided by the total number of pragmatic inferences in the intermediate phase. Results showed that, compared to restudy (*M* = 0.41 ± 0.29), performing a retrieval task during the intermediate phase led to a higher proportion of error correction (*M* = 0.50 ± 0.47, *F*(1,116) = 9.34, *p <* 0.01, *η*^*2*^_*p*_ = 0.07). The presence of feedback led to a much larger proportion of corrected errors (*M* = 0.79 ± 0.27) compared to the absence of feedback (*M* = 0.12 ± 0.13, *F*(1,116) = 468.60, *p <* 0.01, *η*^*2*^_*p*_ = 0.80). But most interestingly, the interaction between the factors (*F*(1,116) = 54.74, *p <* 0.01, *η*^*2*^_*p*_ = 0.32) shows that providing feedback after retrieval resulted in higher error correction (Feedback: *M* = 0.95 ± 0.19; No feedback: *M* = 0.05 ± 0.06; *F*(1,58) = 599.59, *p <* 0.01, *η*^*2*^_*p*_ = 0.91) compared to restudy (Feedback: *M* = 0.63 ± 0.23; No feedback: *M* = 0.19 ± 0.14; *F*(1,58) = 78.30, *p <* 0.01, *η*^*2*^_*p*_ = 0.57, see Fig 1C), suggesting that feedback was more effective in correcting errors after retrieval than after restudy.

### Discussion

Our results showed that retrieval and the presentation of corrective feedback were advantageous tools for learning, even when retrieval was unsuccessful. Compared to restudy, retrieval promoted learning both by perpetuating correct responses and increasing correction of pragmatic inference errors. In the absence of corrective feedback, retrieval led to a higher proportion of false memories compared to restudy, as had already been demonstrated by McDermott [52]. But when feedback was provided, retrieval strengthened error correction in contrast to restudy. The consistent interaction found between learning conditions and feedback suggests that the administration of corrective feedback was more effective when it was presented after retrieval than after restudying practices. These results go in line with an errorful approach to learning and the proposal that after retrieval, memory becomes more amenable to change, promoting memory updating.

However, caution is warranted before further interpretation of our findings since this design had some limitations. In the retrieval condition several types of errors were generated during the intermediate phase (i.e., pragmatic inferences, intrusions, and omissions), while only one type of error was presented in the restudy condition (i.e., pragmatic inferences). Additionally, while at the intermediate phase, 50% of the sentences presented in the restudy condition contained a pragmatic inference error, in the retrieval condition participants committed a pragmatic inference error in 63% of the sentences, leading to an uneven proportion of errors in the two groups, which in turn may have impacted performance at the final cued-recall test. Moreover, in the retrieval condition participants generated false information, whereas those at restudy were presented to it during the experiment which might be a cause for source confusion [31]. Specifically, for those in the retrieval condition, sentences at encoding and as feedback were presented in the experiment setting, coming from an external source, while the pragmatic inferences committed during the intermediate phase were generated internally, coming from an internal source. Yet, for those in the restudy condition, all information (encoding, feedback, and the pragmatic inferences presented during intermediate restudy) was presented in the experiment setting and all came from a shared external source. So, at the final cued-recall test, it is likely that it was more difficult for participants in the restudy condition to identify the source of their memories, than it was for those in retrieval.

To address these limitations, we conducted a second experiment in which we increased the methodological control of the key experimental manipulations during the intermediate phase. To control for the different response types, proportion of responses and the source of the information, we employed a recognition paradigm at the intermediate phase. Participants could engage in one out of two recognition conditions: active or passive recognition. The active recognition condition was analogous to the previous retrieval condition, in which participants had to take a memory test, where they had to decide whether the sentences being presented were correct (like encoding) or incorrect (different from encoding). In contrast, passive recognition was analogous to the previous restudy condition, in which participants were exposed to the same sentences than those at active recognition did but, critically, instead of having to provide an answer, they were presented with the responses given by another participant (from the active condition) and they were instructed only to read the information (i.e., the sentences and the answers given). Therefore, an adapted yoked design was implemented, where the responses of a participant in the active recognition group were presented to another participant in the passive recognition condition, thus forming yoked pairs, and guaranteeing that across the two learning conditions participants were exposed to the same information, in the same proportion, and in the same experimental setting. Presence and absence of corrective feedback was manipulated as in Experiment 1. Following the testing effect and the errorful learning literatures, we predicted that when followed by feedback, the active recognition would lead to better memory performance (i.e., more correct responses) and a higher rate of corrected errors in the final cued-recall test compared to the passive recognition task.

## Experiment 2

### Methods

#### Participants

One hundred and twenty-eight university students from the University of Lisbon participated in this study and received 10€ vouchers as a reward for their time. Because of the COVID-19 pandemic, data collection was conducted online, so participants were asked to evaluate their commitment to the experiment and to report any relevant incidents (see procedure below). Eight participants had to be excluded for the following reasons: 2 reported being interrupted during the experiment, 2 did not have a yoked pair, and 4 reported low levels of attention. Thus, the final sample size included 120 young adults (*M*_age_ = 27.47 ± 8.92; 65 females). Participants were randomly assigned to one out of four conditions from the crossing of the factors, recognition task and feedback: active recognition with feedback (*n* = 31), active recognition without feedback (*n* = 29), passive recognition with feedback (*n* = 31), passive recognition without feedback (*n* = 29). Similarly to Experiment 1, all participants gave their informed consent in the online link of the study. All procedures were approved by the Ethics Committee of the Faculty of Psychology of the University of Lisbon.

#### Materials

Thirty-two pragmatic implication sentences in Portuguese were selected from those adapted by Carneiro et al. [47], and used in Maraver et al. [50] (see S1 Dataset). To improve the experimental control of the materials and to adapt them to a recognition test, sentences were counterbalanced as a function of the format of the sentence presented at encoding (critical vs. filler) and the format presented at the intermediate phase (match vs. mismatch), generating four different sets of the materials. Each participant saw only one set, with the four sets being counterbalanced between participants. In each set, 16 critical sentences were presented at encoding intermixed with 16 fillers. The critical sentences were presented in their original format to generate a pragmatic inference (i.e., “*The karate champion hit the cinder block*”). In contrast, filler sentences (e.g., “*The baby cried all night*”) were created by modifying the critical sentences from another set (e.g., “*The baby stayed awake all night*”), such that they were similar to the critical sentences in all respects, except that the pragmatic implication was explicitly presented in the sentence. By mixing critical and filler sentences we minimized participants’ attention to the inferences that the critical sentences induce and hence prevented participants’ awareness of the experimental manipulation. Afterwards, at the intermediate phase, the presented sentences either matched or mismatched the format of the sentences presented at encoding. A sentence was a match when presented at the intermediate phase in the same format as it was presented at encoding (i.e., “*The karate champion hit the cinder block*”), while a mismatch was a sentence presented at the intermediate phase in a different format from encoding (i.e., “*The karate champion broke the cinder block*”). This complex counterbalance of the material was developed to set a high level of task difficulty, encourage errors to be made, and guarantee that participants would not predict the experimental manipulations. However, because our interest was on the correction of memory errors arising from pragmatic inferences, our analyses focused on the 16 original sentences in which inference generation was promoted.

#### Design

Experiment 2 followed a 2 (recognition: active vs. passive) × 2 (feedback: present vs. absent) design, with both variables manipulated between-participants.

#### Procedure

The experiment was conducted online, and participants completed the experiment in their own computers. They were instructed to complete the study in a calm and quite environment without distractions. Like Experiment 1, the study was programmed and run through the online platform Qualtrics (Qualtrics, Provo, UT).

The structure of the experiment was similar to Experiment 1, except that the learning conditions at the intermediate phase consisted of an active or passive recognition paradigm, instead of a retrieval or restudy tasks (see Table 4). Therefore, during the encoding phase, participants were instructed to read and memorize the 32 sentences (16 critical and 16 fillers) presented at the center of the screen, in black font, for 4.5 seconds. After each sentence they solved a simple math operation for 5 seconds. Sentences were presented in a random order. The key manipulations of the two experimental factors occurred at the intermediate phase. During this phase, in the active recognition condition, participants performed a typical recognition test in which they evaluated whether the sentences presented (in blue font and in a random order) were correct (the same as presented at encoding) or incorrect (different from the sentence presented at encoding). Participants were exposed to each sentence for a minimum of 6.5 seconds and had a maximum of 10 seconds to provide their answer. In the passive recognition condition, participants were exposed to the same sentences (presented in a blue font and in a random order) than those in the active recognition condition but were simultaneously shown the responses given by a previous active participant. Each sentence and corresponding answer were presented for 6.5 seconds, and participants’ task was to read them. Similar to Experiment 1, after giving a response or reading an answer to each sentence at the intermediate phase, the presentation of feedback was manipulated. Half of the participants in the active and passive conditions received corrective feedback that consisted in the presentation of the sentence in the same format as encoding for 4.5 seconds in a black font. The other half did not receive feedback, and after each sentence had 4.5 seconds to solve simple math operations. At the end of the intermediate phase, and after a 5-minutes distractor task, all participants performed a final cued-recall memory test in which they were instructed to complete the missing information in the sentences presented, like in Experiment 1. They were given 60 seconds to complete each sentence.

**Table 4:** Procedure scheme of Experiment 2.

**Encoding phase**				
*The baby stayed awake all night* 5 + 4 = 12				
**Intermediate phase**				
Task	Active recognition	*The baby cried all night* Correct / Incorrect? (Participant gives the response)	Feedback	*The baby stayed awake all night*
			No feedback	23–7 =
	Passive recognition	*The baby cried all night* Correct / **Incorrect** (Participant reads the response)	Feedback	*The baby stayed awake all night*
			No feedback	36 + 6 =
**Final cued-recall test**				
*The baby ____ all night*				

At the end of the study, participants responded to a brief self-report questionnaire about their attention and environmental conditions during the study (similar to Experiment 2 in [50]). Participants were asked to rate their level of attention, the quality of their data, and had a blank space to provide any comments in relation to their performance. After completing this questionnaire, participants were thanked and rewarded for their time.

#### Statistical analyses

Statistical analysis for Experiment 2 focused on the same dependent variables as in Experiment 1. First, we compared recognition memory at the intermediate phase between participants in the feedback and no feedback conditions, through independent samples *t*-tests. Second, we analyzed the different types of response (proportion of correct responses, pragmatic inference errors, intrusions, and omissions) at the final cued-recall test. Lastly, we assessed the memory indexes reflecting persistence of correct responses and error correction. For each dependent variable we conducted an ANOVA with recognition (active vs. passive) and feedback (present vs. absent) as between-subjects factors.

### Results

#### Memory performance at the intermediate phase

At the intermediate phase, the overall accuracy in the active recognition test did not differ between participants who received feedback (*M* = 0.63 ± 0.12) and those who did not (*M* = 0.63 ± 0.08; *t*(53.93) = 0.14, *p* = 0.88, 95% CI [-0.05–0.06]).

#### Memory performance at the final cued-recall test

Descriptive statistics of the different response types assessed at the final cued-recall test as a function of the experimental conditions can be found in Table 3. Results of the between subjects ANOVA showed that participants in the active recognition group had a higher proportion of correct responses than those who completed the passive recognition task at the intermediate phase (*F*(1,116) = 10.91, *p <* 0.01, *η*^*2*^_*p*_ = 0.09). Receiving feedback also generated more correct responses compared to receiving no feedback (*F*(1,116) = 89.68, *p <* 0.01, *η*^*2*^_*p*_ = 0.44). The significant interaction between the factors (*F*(1,116) = 4.42, *p =* 0.04, *η*^*2*^_*p*_ = 0.04) revealed that the beneficial effect of feedback was larger for the active recognition (*F*(1,58) = 95.25, *p <* 0.01, *η*^*2*^_*p*_ = 0.62), than for the passive recognition group (*F*(1,58) = 20.93, *p <* 0.01, *η*^*2*^_*p*_ = 0.26).

For pragmatic inference errors we observed a similar pattern of results. Participants in the active recognition condition had a lower proportion of pragmatic inference errors compared to those in the passive recognition condition (*F*(1,116) = 7.89, *p <* 0.01, *η*^*2*^_*p*_ = 0.06); and receiving feedback led to a decrease in the proportion of pragmatic inferences committed compared to receiving no feedback (*F*(1,116) = 99.99, *p <* 0.01, *η*^*2*^_*p*_ = 0.46). The interaction (*F*(1,116) = 5.87, *p <* 0.02, *η*^*2*^_*p*_ = 0.05) revealed that the beneficial effect of feedback in reducing the proportion of pragmatic inference errors was larger for the active recognition (*F*(1,58) = 122.42, *p <* 0.01, *η*^*2*^_*p*_ = 0.68) than for the passive recognition group (*F*(1,58) = 20.95, *p <* 0.01, *η*^*2*^_*p*_ = 0.26).

No significant effects emerged for intrusion errors (effect of recognition group (*F*(1,116) = 1.18, *p =* 0.28, *η*^*2*^_*p*_ = 0.01); effect of feedback (*F*(1,116) = 0.73, *p =* 0.39, *η*^*2*^_*p*_ = 0.01); interaction (*F*(1,116) = 0.58, *p =* 0.45, *η*^*2*^_*p*_ < 0.01) or for omissions (effect of recognition group *F*(1,116) = 3.65, *p =* 0.06, *η*^*2*^_*p*_ = 0.03); effect of feedback (*F*(1,116) = 1.08, *p =* 0.30, *η*^*2*^_*p*_ = 0.01); interaction (*F*(1,116) = 2.13, *p =* 0.15, *η*^*2*^_*p*_ = 0.02), see Table 3).

#### Persistence of correct responses and error correction

Similar to Experiment 1, we analyzed memory performance at the final cued-recall test as a function of whether the responses were correct or incorrect at the intermediate phase, in order to analyze the persistence of correct responses and error correction.

To analyze the persistence of correct responses, we considered the correct responses at the final cued-recall test that had already been correctly responded/presented at the intermediate phase, divided by the total of sentences correctly recognized/presented at the intermediate phase. Results showed that performing the active recognition test led to a higher persistence of the correct responses (*M* = 0.60 ± 0.28) compared to a passive recognition task (*M* = 0.39 ± 0.29; *F*(1,116) = 20.66, *p <* 0.01, *η*^*2*^_*p*_ = 0.15). Likewise, receiving feedback (*M* = 0.62 ± 0.30) resulted in more persisting correct responses compared to receiving no feedback (*M* = 0.36 ± 0.26, *F*(1,116) = 30.00, *p <* 0.01, *η*^*2*^_*p*_ = 0.21). No significant interaction between the factors was observed (*F*(1,116) = 1.02, *p =* 0.31, *η*^*2*^_*p*_ = 0.01, see Fig 1D).

To assess the correction of errors, we considered the number of sentences that were recognized incorrectly in the intermediate phase but correctly recalled in the final test and divided them by the total number of errors made at the intermediate phase. While no effect of the recognition group was found (*F*(1,116) = 0.44, *p =* 0.51, *η*^*2*^_*p*_ = 0.08), we observed that receiving feedback (*M* = 0.59 ± 0.27) led to a higher rate of error correction than receiving no feedback (*M* = 0.14 ± 0.32; *F*(1,116) = 127.60, *p <* 0.01, *η*^*2*^_*p*_ = 0.52). A significant interaction between the factors (*F*(1,116) = 9.49, *p <* 0.01, *η*^*2*^_*p*_ = 0.07) showed that error correction after feedback compared to no feedback was greater for those in the active recognition condition (Feedback: *M* = 0.67 ± 0.22; No feedback: *M* = 0.09 ± 0.10; *F*(1,58) = 165.92, *p <* 0.01, *η*^*2*^_*p*_ = 0.74), than for those in the passive recognition condition (Feedback: *M* = 0.52 ± 0.30; No feedback: *M* = 0.19 ± 0.20; *F*(1,58) = 24.50, *p <* 0.01, *η*^*2*^_*p*_ = 0.30, see Fig 1F).

#### Discussion

The results extended the findings of Experiment 1 demonstrating the benefit of retrieval in the correction of errors compared to the passive processing of information. Increased methodological control was implemented in this study, since the comparison between conditions relied on a matched proportion of errors between the active and passive recognition tasks as a result of the yoked design. Besides, we replicated the beneficial effect of feedback in the correction of errors when it is presented after an active rather than a passive recognition task. Finally, correct responses persisted to a greater extent for active recognition compared to passive, supporting a consistent testing effect for pragmatic inferences. Taken together, when followed by corrective feedback, unsuccessful testing attempts are useful, and we highlight the consistency of this pattern of results in a highly controlled setting.

## General discussion

In a learning task, we have observed that false memory errors were more easily corrected following the retrieval of a wrong response, than when people were simply exposed to errors. Retrieval seemed to be a more favorable environment to correct errors than the passive processing of information by promoting a better incorporation of the corrective feedback. We have replicated this benefit across two independent studies, both using a cued-recall (Experiment 1) and a recognition paradigm (Experiment 2).

Throughout the two experiments, we have found that responses correctly recalled during the intermediate phase continued to be correctly retrieved at the final memory test, confirming a robust testing effect [4–6]. To the extent of our knowledge, no previous studies have extended the testing effect to pragmatic inference sentences, and we provide additional evidence in support of active memory tests as successful learning events for knowledge acquisition [19]. It is also worth noting that this effect was found even though memory was assessed in an immediate test. The testing effect has been typically found for long term learning, being generally weaker on immediate tests [53], highlighting the robustness of the testing effect.

Besides, as expected, accurate recall was higher when participants received feedback than no feedback, regardless of the learning condition. This corroborates the view that when participants make an error, it is crucial that the correct answer is available to guarantee its processing and integration into memory, rather than simply indicating if the responses were right or wrong [3]. The memory benefit observed following feedback (compared to a no feedback condition) suggests that participants paid attention to the information given, understood that they had made an error, and, as a result, memory was updated, and performance improved. This is consistent with a large body of previous findings [3,8,53], and with the observation that surprising feedback improved memory for the content and its source when presented in response to correct guesses or errors made with high confidence [54]. In our study, we have not compared different features of feedback but we have chosen the parameters that have been proven to be most effective for the correction of false memories, such as explicitly providing the correct answer [18,55], being distinctive [8,17], and appearing immediately after the related false memory [44]. Moreover, future studies should further extend these findings with the current paradigm exploring how the confidence degree of the retrieved errors and the monitoring of the error source modulates its correction. Overall, we provide additional support for the enhanced processing of feedback as a key consequence of error generation [24].

Across two experiments, our main finding was that in the learning process, even when errors occur, retrieving an error benefits subsequent learning compared to the passive processing of the error, as long as corrective feedback is provided. The interaction between the learning conditions and feedback indicates that feedback is more effective when presented immediately after error retrieval than after the passive processing of information. According to the interference account and errorless learning perspective, errors should be avoided during practice to prevent from perpetuating wrongful responses [14]. In contrast, the errorful learning hypothesis states that errors are beneficial for learning as long as feedback is provided, because incorrect responses draw attention to their mismatch from corrective feedback and promote the encoding and incorporation of feedback [15]. Our study was not designed to directly compare the errorless and the errorful learning perspectives, since our focus was on conditions in which errors were present. However, our findings are in line with an errorful approach to learning, since retrieving errors followed by feedback improved later memory performance compared to when no responses were permitted. Although the errorless learning method seems to be helpful for individuals with learning disabilities or amnesia [56, but see 57], our findings build on work suggesting that this does not seem to be the case for healthy adults [58], not even when false memory errors are committed during practice as in the case of the current experiments.

Our results are consistent with previous studies demonstrating that attempting to retrieve information, by itself, enhances learning [19,26]. According to the reconsolidation theory, upon retrieval, memory traces become more labile and prone to modification. Thus, retrieval may facilitate memory updating, but in some cases, retrieval can come with the cost of impairing the learning of new information [59,60]. The Memory Updating After Retrieval (MUAR) framework accounts for the beneficial as well as impairing effects of retrieval on subsequent learning and provides a solid theoretical framework for the interpretation of our findings [22]. In the current studies, we have observed that when relevant information for retrieval (i.e., corrective feedback) is introduced just after a retrieval attempt, there is a ready updating of knowledge. That is, memory malleability after retrieval allows for new related information to be incorporated into the retrieved traces [22]. A previous study using DRM lists has shown that new information presented after retrieval can increase (when false) or reduce (when correct) false memories, and may potentiate error correction, supporting the predictions of the MUAR framework [44]. Our results added evidence to this interpretation since retrieval, compared to the passive processing of information, promoted memory updating through the integration of corrective feedback, as new information aligned with the goals of the retrieval event. The false memory errors generated from pragmatic inferences share close semantic overlap with the correct information, which likely make these errors particularly difficult to notice. Given that pragmatic inference errors stem from semantic association, the benefit of wrongful retrieval to the incorporation of feedback could be explained by a better dissociation between semantic (wrong) and episodic (correct) components, that resulted in a higher rate of correct responses and a lower proportion of pragmatic inferences. Our findings add up to previous research showing that the learning benefit of error generation goes beyond the semantic mediation [28], and we provide additional evidence for active episodic recollection as the underlying mechanism that allows memory updating to learn from errors.

To deepen the study of the mechanisms for error correction, we introduced a manipulation in our second experiment that allowed the comparison between different sources of retrieval. Previous research has shown that being the agent of a retrieved error facilitates memory for the correct answer compared to witnessing other person’s mistake or to a condition when no errors are made [61]. In the current experiments, we used two control conditions (restudy and passive recognition) where errors made by someone else were presented. The detection of an error made by someone else might trigger some degree of unintentional retrieval given that participants were not explicitly told whether or not the response was an error [62,63]. However, the recent study by Carneiro et al., [44] –using a similar restudy condition as our Experiment 1 –did not observe differences in the subsequent retrieval of correct and incorrect sentences that were restudied. Moreover, previous studies have been able to distinguish the retrieval mode from involuntary uses of episodic memory using electrophysiological techniques [64–66], which in addition to Tulving’s proposal that retrieval mode is only engaged through a conscious experience of recollection [41], led us to conclude that it is unlikely that our restudy and passive recognition conditions captured explicit retrieval. Importantly, Metcalfe & Xu suggest that it is the process of overt retrieval of a self-generated error what renders memory open to modification [61]. Our results provide further evidence to this hypothesis since we have observed that being an agent of a retrieved error and receiving feedback facilitated memory for the correct answer more than witnessing an error committed by someone else and receiving feedback afterwards. In our case this comparison was made between-groups, so it still remains unexplored whether the benefits of self-generating an error compared to seeing someone else’s mistake and then receiving feedback are maintained within the same participants. The comparison between errors from different sources provides an additional argument in support of the benefits of retrieval for learning, since it shows that it is not only the mere exposure to an error followed by its correction that promotes learning. Instead, what underlies the full benefit of learning from errors is the retrieval of an own error which renders the exposed memory trace vulnerable to modification by corrective feedback [3,61].

The current study is not exempt of limitations. Importantly, the assessment of memory performance was done immediately after, representing a measure of short-term learning. Future studies should explore whether this benefit of retrieval for the correction of errors still holds after long delays and are beneficial for long-term learning. False memories represent a particularly interesting type of error for the study of error correction, since they are normally held with high levels of confidence [67–69]. Fazio & Marsh observed a hypercorrection effect for pragmatic inferences so that errors made with higher levels of confidence were easier to correct than low confidence errors [70]. In the current study, the levels of confidence were not assessed, so it remains unexplored whether this hypercorrection effect is replicated across different learning conditions [69].

Caution is warranted when generalizing our findings to the classroom given that our materials were developed to generate a high proportion of errors and differ from educational environments where errors of different nature coexist (e.g., omissions in addition to retrieval failures). However, our results show that testing is a powerful mean to improve learning, not just assessing it. Making errors in tests and during the process of learning derives direct benefits to learners, when followed by feedback that offers the correct response. Thus, we support the use of learning strategies where errors are tolerated, and students engage in active, exploratory, and generative learning. Going a step forward, recent research suggests that learning can benefit from errors even when participants are aware of the correct answer and deliberately decide to commit an error, in contrast to avoiding giving incorrect answers, an effect called the *derring effect* with important implications for educational practice given that students often appear to be unaware of the benefits of deliberate erring [71]. In comparison with approaches that stress error avoidance, making training more challenging by allowing false starts and errors followed by feedback, discussion, and correction may ultimately lead to better and more flexible transfer of skills to later critical situations.

## Conclusion

Our results suggest that the act of retrieval constitutes a valuable learning event. Even when the information is wrong, retrieval does more than correcting wrong information, and it is through the effortful engagement in intentional explicit retrieval what renders memory in a sufficient malleable state to allow the incorporation of feedback.

We propose that the benefits of retrieval go beyond the testing effect given that retrieval also facilitated false memory correction. Our results support the use of tests and active retrieval strategies in learning, showing we can learn from practice even when errors are generated.

## Supporting information

S1 Dataset The datasets presented in this study can be found in the following Open Science Framework project identifier: DOI 10.17605/OSF.IO/JVU4K. (DOCX)
