# Supplementary material for: Can we learn from errors? Retrieval facilitates the correction of false memories for pragmatic inferences
Source: PLoS One. 2022 Aug 2;17(8):e0272427. doi: 10.1371/journal.pone.0272427 (PMC9345471; doi:10.1371/journal.pone.0272427)
Supplement: S1 Dataset — (DOCX) [file pone.0272427.s001.docx]

**Supplementary Material 1.** Thirty-two pragmatic inference sentences used as experimental material. For each sentence, the correspondent responses that were coded as correct or as a pragmatic inference error are indicated.

| **Sentence** | **Correct Response** | **Pragmatic Inference** |
| --- | --- | --- |
| After dropping off her kids at the school, the mother picked up bread. | picked up | bought |
| The charming prince gently put his lips towards Snow White’s cheek. | put his lips towards/approached | kissed |
| The snowman vanished when the temperature reached 26ºC. | vanished/disappeared | melted |
| The absent-minded professor didn’t have his car keys. | didn't have | forgot |
| The friendly bartender got extra money from the customer. | extra money/some coins | tips |
| The angry rioter took aim with the stone at the window. | took aim/hit the bull's eye | threw |
| That radio station liked only hard rock music. | liked | played |
| The karate champion hit the cinder block. | hit | broke/smashed/split |
| Dennis the Menace sat in Santa’s chair and asked for an elephant. | chair | lap |
| The painter knocked over the bucket of black paint. | knocked over | spilled/threw |
| The hungry python caught the mouse. | caught | ate |
| The hungry squirrel was biting the grass | was biting | was eating |
| The safe-cracker put the match to the fuse. | put the match to | lit |
| The agile cat reached the fish with its claws. | reached | caught |
| The rat was attracted by the mousetrap. | attracted | caught |
| Ricardo just wanted to rest for a whole night. | rest | sleep |
| As soon as she reached the beach, Martha spread the towel, put on her sun hat, and lay down on the sand. | sun hat | sunscreen |
| The night watchman took some coffee from his thermos. | took | drank |
| King Kong stood on top of the Empire State Building. | stood on top of | climbed |
| The new baby stayed awake all night. | stayed awake | cried |
| The noisy guard dog growled at the trespasser. | growled | barked |
| The hypnotist put his fingers together and awakened his client | put his fingers together | snapped his fingers |
| The Christian closes his eyes and remains a few minutes in silence before each meal. | remains a few minutes in silence | prays |
| The thief entered a store and took a chocolate bar. | took | stole |
| In the middle of the night the sleepy husband went to get the newspaper and hit the mosquito. | hit | killed |
| The race started when the referee pulled the trigger. | pulled the trigger | fired the gun |
| After chasing the thief for three blocks, the cop finally reached the thief. | reached | caught |
| The kid stung the balloon with a pin. | stung | popped/burst |
| The captain of the submarine said: -'Submerge submarine!' | said | ordered |
| She picked up her favorite pair of shoes and left. | picked up | put on/wore |
| He picked up the phone, seeing that it was his mother who was calling. | picked up | answered |
| On the day of the interview, she chose her lucky shirt. | chose | wore |
